# ERCP-Related Adverse Events in Primary Sclerosing Cholangitis: A Systematic Review and Meta-Analysis

**DOI:** 10.1155/2022/2372257

**Published:** 2022-07-21

**Authors:** Navneet Natt, Faith Michael, Hope Michael, Sacha Dubois, Ahmed Al Mazrou'i

**Affiliations:** ^1^Northern Ontario School of Medicine University, Sudbury and Thunder Bay, Ontario, Canada; ^2^McMaster University, Hamilton, Ontario, Canada; ^3^Centre for Applied Health Research, St. Joseph's Care Group, Thunder Bay, Ontario, Canada; ^4^School of Nursing, Faculty of Health and Behavioural Sciences, Lakehead University, Thunder Bay, Ontario, Canada; ^5^Department of Gastroenterology, Health Sciences North, Sudbury, Ontario, Canada

## Abstract

**Background and Aims:**

While endoscopic retrograde cholangiopancreatography (ERCP) is a valuable diagnostic and therapeutic tool in primary sclerosing cholangitis (PSC), there is conflicting data on associated adverse events. The aims of this systematic review and meta-analysis are to (1) compare ERCP-related adverse events in patients with and without PSC and (2) determine risk factors for ERCP-related adverse events in PSC.

**Methods:**

Embase, PubMed, and CENTRAL were searched between January 1, 2000, and May 12, 2021. Eligible studies included adults with PSC undergoing ERCP and reported at least one ERCP-related adverse event (cholangitis, pancreatitis, bleeding, and perforation) or an associated risk factor. The risk of bias was assessed with the Newcastle-Ottawa scale and Cochrane Risk of Bias 2. Raw event rates were used to calculate odds ratios (ORs) and then pooled using random-effects models.

**Results:**

Twenty studies met eligibility criteria, of which four were included in a meta-analysis comparing post-ERCP adverse events in patients with PSC (*n* = 715) to those without PSC (*n* = 9979). We found a significant threefold increase in the 30-day odds of cholangitis in PSC compared to those without (OR 3.263, 95% CI 1.076–9.896; *p*=0.037). However, there were no significant differences in post-ERCP pancreatitis (PEP), bleeding, or perforation. Due to limitations in primary data, only risk factors contributing to PEP could be analyzed. Accidental passage of the guidewire into the pancreatic duct (OR 7.444, 95% CI 3.328–16.651; *p* < 0.001; *I*^2^ = 65.0%) and biliary sphincterotomy (OR 4.802, 95% CI 1.916–12.033; *p*=0.001; *I*^2^ = 73.1%) were associated with higher odds of PEP in a second meta-analysis including five studies.

**Conclusions:**

In the context of limited comparative data and heterogeneity, PSC patients undergoing ERCP have higher odds of cholangitis despite the majority receiving antibiotics. Additionally, accidental wire passage and biliary sphincterotomy increased the odds of PEP. Future studies on ERCP-related risks and preventive strategies are needed.

## 1. Introduction

Primary sclerosing cholangitis (PSC) involves inflammation of the biliary tract resulting in fibrosis of the intra- and/or extrahepatic ducts [1, 2]. This condition is associated with complications including bile duct stenoses, recurrent cholangitis, cirrhosis, and hepatobiliary malignancies [1, 2]. Endoscopic retrograde cholangiopancreatography (ERCP) remains a valuable diagnostic and therapeutic tool in PSC [1–3]. It provides the opportunity for biopsy when imaging is inconclusive and to exclude sequelae such as cholangiocarcinoma [3]. Furthermore, ERCP allows for therapeutic interventions to relieve biliary obstruction [3].

Although ERCP plays an important role, it is not without potential risks, which include pancreatitis, bleeding, cholangitis, and perforation [4, 5]. Small retrospective studies have reported high rates of cholangitis in patients with PSC [6]. Accordingly, clinical practice guidelines recommend antibiotics in PSC to prevent post-ERCP cholangitis, but this is based on low-quality evidence [2, 4, 7, 8]. Few studies have compared the rates of ERCP adverse events in those with and without PSC [8–10]. Furthermore, no systematic review has examined the risk factors for post-ERCP adverse events in PSC.

The objectives of this systematic review and meta-analysis are (1) to compare the odds of ERCP-related adverse events in patients with PSC to those without and (2) to identify risk factors for the development of ERCP-related adverse events in PSC.

## 2. Methods

### 2.1. Registration

The protocol for this study was prospectively registered on PROSPERO (CRD42021281265) and developed in accordance with the Preferred Reporting Items for Systematic Reviews and Meta-Analyses (PRISMA) guidelines (Supplemental Figure S1).

### 2.2. Information Sources and Search Strategy

A systematic literature search was conducted in Embase, PubMed, and Cochrane Central Register of Controlled Trials (CENTRAL) for studies published from January 1, 2000, to May 12, 2021. The search strategy was guided by a research librarian and consisted of three components: (1) PSC, (2) ERCP, and (3) adverse events. The Embase search strategy is available in Supplemental Figure S2. The strategy was adapted for the other databases. Language restrictions were not applied. Reference lists of relevant articles, including systematic reviews and meta-analyses, were hand searched. The grey literature was searched through Scopus, OpenGrey, ClinicalTrials.gov, manual web searches, and conference proceedings.

### 2.3. Eligibility Criteria and Selection Process

Eligible studies included adults (age ≥18 years) with PSC undergoing ERCP and reported at least one ERCP-related adverse event (cholangitis, pancreatitis, bleeding, and perforation) or analyzed risk factors for developing an ERCP-related adverse event. Studies eligible for inclusion in the systematic review were also assessed for inclusion in meta-analyses. Studies that compared ERCP-related adverse events in patients with PSC to those without PSC were considered for inclusion in the first meta-analysis while those reporting risk factors for an adverse event in patients with PSC were considered for inclusion in a separate meta-analysis. Cohort studies and randomized controlled trials (RCTs) were included. Case reports, case series, systematic reviews, meta-analyses, and clinical guidelines were excluded. Studies published prior to 2000 were excluded due to more recent advances in ERCP that could affect data interpretation.

Titles and abstracts were independently reviewed in duplicate for inclusion followed by a full-text review (N. N., F. M., H. M.). Disagreements were resolved by discussion or in consultation with a fourth author (A. A.).

### 2.4. Data Collection and Synthesis

The following data was extracted in duplicate by two investigators (N. N., F. M.): study design, study period, sample size, patient characteristics (age, sex, PSC characteristics, comorbidities, and previous ERCPs), procedure characteristics (indication, duration, intraprocedural interventions, endoscopist experience, and periprocedural antibiotics), outcome definitions, and outcome data (30-day rates of cholangitis, pancreatitis, bleeding, and perforation).

#### 2.4.1. Objective 1: ERCP-Related Adverse Events

The first meta-analysis compared ERCP-related adverse events in those with and without PSC. The outcomes of interest were 30-day cholangitis, pancreatitis, bleeding, and perforation.  Table 1 summarizes the definitions of outcomes where provided by the included studies. The meta-analysis was complemented by a narrative synthesis of the remaining noncomparative studies.

Analyses were completed by a research statistician (S. D.) using Stata's metan command [11, 12]. Raw event rates for adverse events were used to calculate odds ratios (ORs) and standard errors. Outcomes were pooled using a random-effects model described by DerSimonian and Laird, and 95% confidence intervals (CIs) were reported for summary estimates [13]. The *I*^2^ was computed to examine heterogeneity. An *I*^2^ >50% was considered to represent substantial heterogeneity. A *p* value of <0.05 was considered to be statistically significant. Results were presented in forest plots. A sensitivity analysis was conducted to explore the impact of excluding Adler et al. as this study specifically included patients with PSC and cirrhosis.

#### 2.4.2. Objective 2: Risk Factors for ERCP-Related Adverse Events

A second meta-analysis included studies that analyzed risk factors for ERCP-related adverse events in patients with PSC. Due to a lack of primary data, only risk factors for post-ERCP pancreatitis (PEP) were amenable to a meta-analysis. Methods for this analysis were the same as above [11–13]. However, unadjusted ORs were extracted from primary studies, instead of event rates, and pooled using a random-effects model as described previously. Results were presented in forest plots and accompanied by a narrative synthesis.

### 2.5. Quality Assessment

The risk of bias of nonrandomized comparative studies was assessed using the Newcastle-Ottawa scale [14]. RCTs were assessed using the Cochrane Risk of Bias 2 tool [15]. Two investigators (N. N., F. M.) independently assessed each study. Disagreements were resolved through discussion, and when required, in consultation with a third investigator (A. A.). Certainty of the evidence was assessed using the Grading of Recommendations, Assessment, Development, and Evaluations (GRADE) framework [16]. Heterogeneity was assessed with a sensitivity analysis that excluded Adler et al. as described above.

## 3. Results

### 3.1. Study Selection

**Figure 1:**
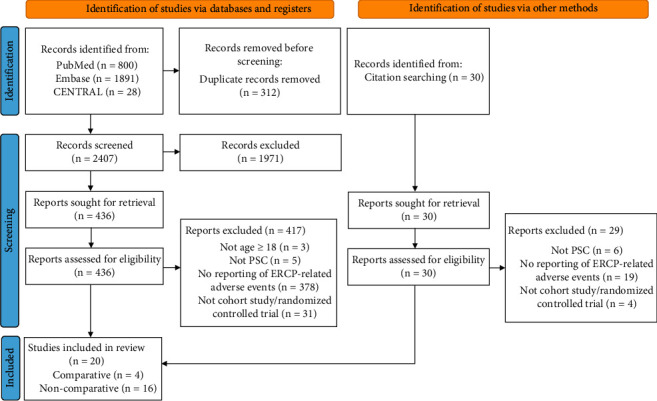
Flow diagram of study selection according to Preferred Reporting Items for Systematic Reviews and Meta-Analyses standards.

The literature search yielded 2749 studies (800 from PubMed, 1891 from Embase, 28 from CENTRAL, and 30 from searching reference lists) (Figure 1). Duplicates were removed and 1971 studies were excluded through the title and abstract review. The remaining 466 studies were reviewed in full text. Twenty studies met the inclusion criteria for this systematic review [8, 9, 17–34]. Of those, four studies were identified that compared ERCP-related adverse events in patients with PSC to those without PSC and were amenable to a meta-analysis [8, 9, 17, 18]. We further identified five studies, among the initial twenty, which reported risk factors for ERCP-related adverse events and included these in a separate meta-analysis [25, 27, 28, 31, 32].

### 3.2. Objective 1: ERCP-Related Adverse Events

#### 3.2.1. Characteristics of Included Studies and Patients

Twenty studies were included, which consisted of 3886 patients with PSC and 9979 patients without PSC. The average age ranged from 35 years to 50 years in PSC and 56 years to 69 years in non-PSC (Table 2). Information on the performed ERCPs was inconsistently reported (Table 3). Overall, there were high rates of intervention, such as stent insertions and sphincterotomies. The majority of patients received periprocedural antibiotics for cholangitis prophylaxis.

The four comparative studies that contributed to the adverse events meta-analysis were retrospective cohorts and included 715 patients with PSC and 9979 patients without PSC [8, 9, 17, 18]. The risk of bias was assessed with the Newcastle-Ottawa scale, where a maximum of 9 points can be awarded (Table 4). Three studies [8, 9, 18] received 6 points, while the remaining study [17] received 5 points.

#### 3.2.2. Cholangitis

**Figure 2:**
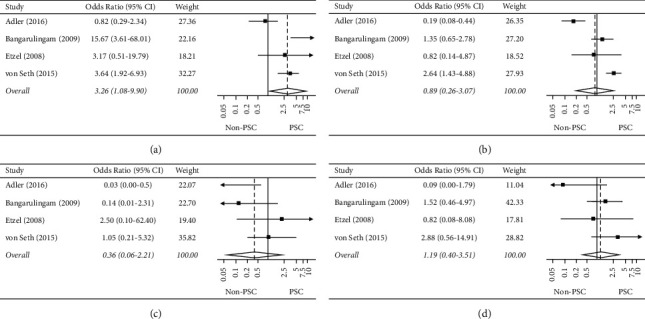
(a) Forest plot of the individual and combined odds of cholangitis, (b) forest plot of the individual and combined odds of pancreatitis, (c) forest plot of the individual and combined odds of bleeding, and (d) forest plot of the individual and combined odds of perforation.

Eighteen studies reported on cholangitis with rates varying between 1% and 7.9% [8, 9, 17–20, 22–33]. Four of these studies compared patients with and without PSC [8, 9, 17, 18]. On meta-analysis of these studies, there was a significant threefold increase in the odds of 30-day cholangitis in those with PSC compared to those without (4.3% vs. 2.0%; OR 3.263, 95% CI 1.076–9.896; *p*=0.037; *I*^2^ = 73.0%; Figure 2(a)) [8, 9, 17, 18].

Eighteen studies reported on antibiotic use for cholangitis prophylaxis in PSC (Table 3) [8, 9, 17–20, 22–33]. The most commonly used antibiotics included cephalosporins and fluoroquinolones [19, 20, 25–27, 31–33]. In twelve studies, antibiotics were given at the time of ERCP or just prior to the procedure [8, 17–19, 22–27, 30, 33]. In five studies, patients received both prophylactic and postprocedural antibiotics for a duration ranging from 24 hours to 5 days [20, 28, 29, 31, 32]. In the one remaining study, antibiotics were left to the discretion of the endoscopist [9]. In sixteen studies, the use of prophylactic antibiotics exceeded 95% [8, 9, 17, 20, 22–33]. Von Seth et al. reported the lowest use of prophylactic antibiotics (49%) [18] followed by Alkhatib et al. (77%) [19].

Nine studies reported on the management of cholangitis [19, 20, 22, 24, 26, 28–30, 33]. Nearly all patients were managed medically and there was no associated mortality. In four studies, patients were managed with antibiotics only [20, 26, 30, 33]. In another four studies, patients required endoscopic intervention [19, 24, 28, 29]. Only one study reported a severe infection that required surgical intervention [22].

#### 3.2.3. PEP

The PEP event rate ranged from 0% [30] to 14.3% [32] across all twenty studies [8, 9, 17–34] with most studies reporting a PEP rate of 5% or less [8, 9, 17–24, 26, 28–31, 33, 34]. On meta-analysis of the four comparative studies, there was no significant difference in 30-day PEP between those with and without PSC (4.2% vs. 3.4%; OR 0.888, 95% CI 0.257–3.069; *p*=0.851; *I*^2^ = 87.9%; Figure 2(b)) [8, 9, 17, 18].

Six studies reported on the severity of PEP in 172 patients with PSC [19, 20, 22, 25, 27, 34]. Fifty-two cases of PEP (30.2%) were deemed moderate to severe. Two studies compared the severity of PEP in those who received rectal nonsteroidal anti-inflammatory drugs (*n* = 50) to those who did not receive rectal NSAIDs (*n* = 70) [27, 34]. Overall, 12.9% of PSC patients without rectal NSAIDs developed severe pancreatitis compared to 10% of PSC patients who did receive rectal NSAIDs (*p* value not reported). Of the seven studies that described the management of PEP, all cases (*n* = 70) were managed conservatively, with the exception of one patient who required surgical debridement [20, 24–26, 28, 29, 33]. Two studies reported on the average length of hospitalization after PEP, and this was 3.5 [28] and 4 [25] days, respectively.

#### 3.2.4. Bleeding

Bleeding among PSC patients varied from 0% to 3.2% across all twenty studies [8, 9, 17–34]. On meta-analysis of the four comparative studies, odds of bleeding were similar in those with PSC compared to those without (0.3% vs. 1.1%; OR 0.363, 95% CI 0.060–2.214; *p*=0.272; *I*^2^ = 50.3%; Figure 2(c)) [8, 9, 17, 18].

The management of post-ERCP bleeding was only described for eleven patients across three studies [26, 28, 29]. Most bleeding events were self-limited with only two patients requiring blood transfusion [28, 29]. Endoscopy was required in three patients, with one requiring intensive care unit admission [28]. There was no associated mortality due to bleeding.

#### 3.2.5. Perforation

Sixteen studies reported on perforation after ERCP in patients with PSC [8, 9, 17–21, 23–27, 29, 31–33]. Most studies found rates between 0% and 2%. On meta-analysis of the four comparative studies, odds of perforation were similar between groups (0.7% PSC vs. 0.5% non-PSC; OR 1.191, 95% CI 0.402–3.515; *p*=0.752; *I*^2^ = 28.5%; Figure 2(d)) [8, 9, 17, 18].

Four studies presented management in nine patients who developed post-ERCP perforation [24–26, 33]. Only one [24] required surgical drainage for a bile leak, while all other cases were managed conservatively [25, 26, 33].

#### 3.2.6. Sensitivity Analysis

Since results were limited by heterogeneity, a sensitivity analysis was conducted to explore the effects of excluding Adler et al. Distinct from the other studies, Adler et al. only included PSC patients with cirrhosis [17]. Accordingly, heterogeneity was considerably reduced with the removal of Adler et al. (Supplemental Figure S3). The odds of 30-day cholangitis remained elevated among PSC patients compared to those without (OR 5.159, 95% CI 2.080–12.796; *p* < 0.001; *I*^2^ = 39.9%) [8, 9, 18]. Pancreatitis reached statistical significance with a *p* value of 0.049 (OR 1.794, 95% CI 1.002–3.214; *I*^2^ = 29.3%). Bleeding (OR 0.782, 95% CI 0.207–2.959; *p*=0.718; *I*^2^ = 3.8%) and perforation (OR 1.666, 95% CI 0.686–4.046; *p*=0.259; *I*^2^ = 0.0%) were similar between the two groups, which is consistent with the original analysis.

### 3.3. Objective 2: Risk Factors for PEP

#### 3.3.1. Characteristics of Included Studies and Patients

Due to limitations in primary data for cholangitis, bleeding, and perforation, only risk factors contributing to PEP in PSC could be assessed. Three retrospective cohorts [25, 28, 31], one retrospective case-control [27], and one RCT [32] contributed to this meta-analysis. In total, 1939 patients with PSC were included, of which 61.1% were male (*n* = 1185) (Tables 2 and 3). Risk factors were female sex, accidental guidewire passage into the pancreatic duct (PD), and biliary sphincterotomy.

The risk of bias among nonrandomized studies was assessed with the Newcastle-Ottawa scale (Tables 4 and 5). One study [31] received 8 points, while the remaining three studies [25, 27, 28] received 6 or less points. The included RCT [32] was assessed with the Cochrane Risk of Bias 2 tool (Table 6) and was assessed to have some concerns.

#### 3.3.2. Female Sex

**Figure 3:**
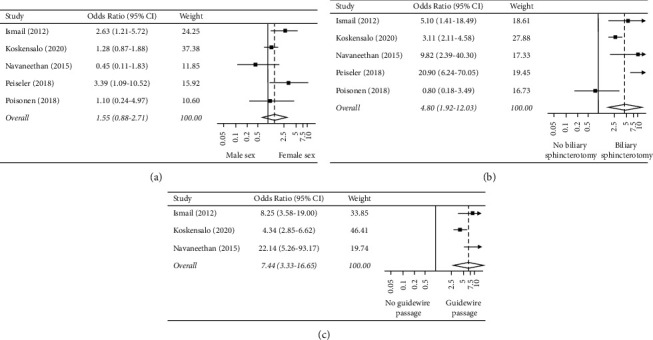
Forest plot of the individual and combined odds of pancreatitis based on (a) sex, (b) biliary sphincterotomy, and (c) accidental passage of the guidewire into the pancreatic duct.

The association between female sex and PEP was explored in five studies [25, 27, 28, 31, 32]. Female sex was associated with increased PEP in patients with PSC on multivariate analyses by Ismail et al. [25] (OR 2.4, 95% CI 1.0–5.8, *p* = 0.046) and Peiseler et al. [31] (OR 3.57, 95% CI 1.2–10.6, *p* = 0.022). However, in three different studies [27, 28, 32], there was no significant association between sex and PEP. Similarly, in the meta-analysis of all five studies, female sex was not associated with PEP (OR 1.546, 95% CI 0.882–2.709; *p* = 0.128; *I*^2^ = 47.6%; Figure 3(a)).

#### 3.3.3. Biliary Sphincterotomy

Biliary sphincterotomy at the current ERCP was examined in five studies [25, 27, 28, 31, 32]. Overall, in the meta-analysis, biliary sphincterotomy was associated with a nearly fivefold increase in the odds of PEP (OR 4.802, 95% CI 1.916–12.033; *p*=0.001; *I*^2^ = 73.1%; Figure 3(b)).

#### 3.3.4. Accidental Guidewire Passage

The association of PEP with accidental guidewire passage into the PD was examined in three studies [25, 27, 28]. Two of these found that guidewire passage into the PD increased PEP (Navaneethan: OR 22.14, 95% CI 5.26–93.15; *p* < 0.001; [28] Koskensalo: OR 1.876, 95% CI 1.059–3.322; *p*=0.031 [27]). Ismail et al. also demonstrated an association, in particular, that the incidence of PEP increased with the number of guidewire passes [25]. The incidence of PEP was 2.6% in cases without passage into the PD, 20% when there were two passes, and 31.6% when there were five passes into the PD (*p* < 0.001) [25]. In the meta-analysis of these three studies, the accidental passage of the wire into the PD was associated with a sevenfold increase in the odds of PEP (OR 7.444, 95% CI 3.328–16.651; *p* < 0.001; *I*^2^ = 65.0%; Figure 3(c)).

## 4. Discussion

Our systematic review and meta-analyses have demonstrated the following important findings: (1) PSC patients have higher odds of post-ERCP cholangitis, but similar odds of PEP, bleeding, and perforation as those without PSC, and (2) risk factors for the development of 30-day PEP in PSC include guidewire passage into the PD and biliary sphincterotomy but not female sex (Table 7).

### 4.1. Cholangitis

The natural history of PSC involves the formation of biliary strictures which predisposes to cholangitis. ERCP is often performed to relieve biliary obstruction in this setting. However, ERCP can simultaneously be a risk factor for postprocedural cholangitis, which may be related to chronic bacterial colonization [2, 4]. As such, antibiotic prophylaxis is recommended to prevent post-ERCP cholangitis in this population; however, this recommendation is based on low-quality evidence [2, 7]. Our review provides evidence to support this guideline recommendation by confirming that the 30-day odds of cholangitis are higher in patients with PSC compared to those without PSC (4.3% vs. 2.0%; OR 3.263, 95% CI 1.076–9.896; *p*=0.037). Based on the sensitivity analysis, the odds of cholangitis in PSC could be as high as fivefold the odds of those without PSC (OR 5.159, 95% CI 2.080–12.796; *p* < 0.001; *I*^2^ = 39.9%). This was despite consistent antibiotic use in most studies, which mirrors previous research demonstrating difficulties eradicating bacterial overgrowth in PSC patients [35]. Notably, antibiotic type, route of administration, and duration varied considerably between studies, and currently, there are no randomized studies to guide optimal antibiotic treatment [2].

In addition to antibiotics, disease phenotype may also affect the risk of post-ERCP cholangitis. For example, Ponsioen et al. conducted a multicenter RCT and reported the highest rate of cholangitis at 7.9%, despite all patients receiving prophylactic antibiotics two hours prior to ERCP and 24 hours after the procedure [32]. Of note, this study was conducted in PSC patients with dominant strictures (DS), which cause obstruction and predispose to cholangitis.

Unfortunately, few studies explored risk factors for post-ERCP cholangitis in patients with PSC, limiting our ability to conduct a meta-analysis. Future studies should explore risk factors, such as biliary stenting, for the development of post-ERCP cholangitis, and provide strategies for risk modification in this vulnerable population.

### 4.2. PEP

PEP is a well-recognized complication of ERCP, and our original analysis demonstrated no statistically significant difference in the odds of 30-day PEP between patients with and without PSC (4.2% vs. 3.4%; OR 0.888, 95% CI 0.257–3.069; *p* = 0.851; *I*^2^ = 87.9%). Interestingly, when Adler et al. was excluded in the sensitivity analysis, we found a statistically significant increase in the odds of PEP among patients with PSC (OR 1.794, 95% CI 1.002–3.214; *p* = 0.049; *I*^2^ = 29.3%). Previous research has demonstrated morphologic pancreatic ductal changes in up to 24% of patients with PSC which may predispose them to inflammation and PEP [36]. The high rate of endoscopic intervention in PSC may also contribute to increased PEP in this population. For example, the study with the highest reported incidence of PEP involved high rates of therapeutic intervention for DS [32].

Female sex has been associated with the development of PEP in those without PSC, although the underlying mechanism remains unclear [4, 37, 38]. Difficult cannulation and increased incidence of the sphincter of Oddi dysfunction have been postulated as possible contributing factors [39]. In this meta-analysis, female sex was not associated with increased PEP in patients with PSC (OR 1.546, 95% CI 0.882–2.709; *p* = 0.128; *I*^2^ = 47.6%).

In this review, biliary sphincterotomy was associated with higher odds of PEP in patients with PSC (OR 4.802, 95% CI 1.916–12.033; *p*=0.001; *I*^2^ = 73.1%). This mirrors previous findings in those without PSC [40]. It is believed that electrocautery during sphincterotomy causes thermal injury and edema to the surrounding tissues, precipitating pancreatitis. It is likely that a similar mechanism ensues in PSC patients [41].

Accidental guidewire passage into the PD also predicted the development of PEP in patients with PSC (OR 7.444, 95% CI 3.328–16.651; *p* < 0.001; *I*^2^ = 65.0%). In patients without PSC, increased pressure and irritation to the PD from guidewire placement have been implicated as the underlying mechanism, and we hypothesize that a similar phenomenon occurs in PSC [42].

Prevention of PEP has been explored in the general population in the form of rectal nonsteroidal anti-inflammatory drug (NSAIDs) administration, intensive fluid hydration, and prophylactic pancreatic stent placement, among other prophylaxis strategies [43–45]. In our systematic review, two studies explored the role of rectal NSAIDs in PSC and presented discrepant results, thus making this an area for further research in patients with PSC [27, 34]. Future research should also explore the impact of other prophylactic strategies to minimize the incidence and/or severity of PEP in patients with PSC.

### 4.3. Bleeding and Perforation

In this systematic review, bleeding and perforation were relatively uncommon adverse events that occurred in less than 2% of PSC patients across included studies. There were no statistically significant differences in the 30-day odds of bleeding (0.3% vs. 1.1%; OR 0.363, 95% CI 0.060–2.214; *p*=0.272; *I*^2^ = 50.3%) or perforation (0.7% vs. 0.5%; OR 1.191, 95% CI 0.402–3.515; *p*=0.752; *I*^2^ = 28.5%) between those with and without PSC. This remained the case on sensitivity analysis.

Both bleeding and perforation often occurred in the setting of endoscopic intervention and were managed conservatively in most cases [20, 21, 26, 28, 29]. The study with the highest rate of post-ERCP perforation at 15.9% involved balloon dilation in 96.8% of patients (*n* = 61) and temporary stent insertion in 52% of patients (*n* =33) [20]. Similarly, the two studies that reported the highest rates of post-ERCP bleeding occurred in the context of sphincterotomy [21] or therapeutic intervention for DS [26].

Overall, the incidences of bleeding and perforation were similar to those without PSC, which is not unexpected as the pathophysiology of PSC would not modulate these adverse events [6]. Furthermore, the lack of severe complications could in part be explained by the relatively young and otherwise healthy population. For example, patients with PSC tend to be free of comorbidities that would require antithrombotic therapy and increase bleeding risk.

### 4.4. Strengths and Limitations

To our knowledge, this is the first systematic review and meta-analysis to examine post-ERCP adverse events in patients with PSC and analyze associated risk factors. This review included data from 3886 patients with PSC across twenty studies. We developed a broad search strategy and included grey literature to conduct the most comprehensive literature search on this topic to date. Additionally, this is a clinically relevant topic as post-ERCP adverse events are important for clinicians to predict and counsel patients on, especially in high-risk populations, such as PSC.

The limitations of our review are related to the primary studies. All but one of the included studies were observational in design, with most being retrospective (Table 2). There was also a lack of data on certain topics that limited the potential for additional meta-analyses. For example, while we identified a statistically significant increase in the odds of post-ERCP cholangitis in PSC, we were unable to evaluate risk factors for developing this adverse event. Furthermore, there was inconsistent reporting of patient and procedural characteristics, which would aid in the contextualization of results. Finally, studies varied in the characteristics of included patients; therefore, results should be interpreted in the context of heterogeneity.

### 4.5. Implications for Practice

Patients with PSC frequently undergo multiple ERCPs throughout their lifetime to relieve biliary obstruction, perform therapeutic interventions, and exclude associated sequelae. Traditionally, patients with PSC have been deemed high-risk for post-ERCP adverse events, especially cholangitis, though there is limited evidence to support this notion.

This systematic review and meta-analysis has important practice implications. We identified a statistically significant increase in odds of cholangitis in PSC. This was despite the use of prophylactic antibiotics in most included patients, as recommended by current guidelines. This highlights the need to review current antibiotic practices and determine additional strategies to reduce the risk of cholangitis in this high-risk population. Future research should also explore risk factors in the development of cholangitis in patients with PSC. For example, PSC is often associated with autoimmune hepatitis and inflammatory bowel disease—conditions that warrant treatment with immunosuppressants, increasing the risk of infection. This information was inconsistently reported and may be an avenue for further investigation.

This systematic review also demonstrated that patients with PSC are at similar risk of other adverse events, such as bleeding, perforation, and pancreatitis as those without PSC. This can inform patient counseling on periprocedural ERCP risks. Furthermore, when examining risk factors for the development of PEP, it was noted that patients with PSC shared many of the same risk factors as those already established in patients without PSC, such as accidental guidewire passage into the PD and biliary sphincterotomy. Further research is needed to explore risk factors for other ERCP-related adverse events in this vulnerable population.

## 5. Conclusion

In summary, patients with PSC have a higher odds of post-ERCP cholangitis than those without PSC. We found similar odds of PEP, bleeding, and perforation between the two groups. While the analysis of risk factors for post-ERCP adverse events was limited to that of PEP, risk factors among patients with PSC appear to mirror that of the general population. Further research is required to determine strategies to mitigate ERCP-related adverse events as this remains a valuable diagnostic and therapeutic entity.

## Figures and Tables

**Table 1:** Definition of ERCP-related adverse events.

	Cholangitis	Pancreatitis	Bleeding	Perforation
Adler (2016)	Not defined	Not defined	Not defined	Not defined
Bangarulingam (2009)	Requiring hospitalization for intravenous/intramuscular antibiotics for fever within 2 days of the procedure	Admission to hospital with abdominal pain and documentation of pancreatitis by the gastroenterologist	Hospitalization within 1 week of procedure with melena requiring transfusion or confirmation of bleeding on repeat endoscopy	Documentation of contrast extravasation during the procedure by the endoscopist, or evidence of bile leak on abdominal imaging after the procedure
Etzel (2008)	Not defined	Not defined	Not defined	Not defined
Von Seth (2015)	Defined at the discretion of the attending clinician in patients requiring hospitalization for intravenous antibiotics	Abdominal pain and elevation in amylase by at least three times more than 24 hours from the procedure, and requiring admission or prolongation of admission for at least 2 days	Confirmed evidence of bleeding requiring transfusion and/or endoscopic/surgical intervention	Bowel or bile duct perforation

**Table 2:** Characteristics of included studies and patients.

	Study design	Number of patients	Number of ERCPs	Age (years)	Male *n* (%)	PSC characteristics	Relevant comorbidities
Adler (2016)	Retrospective cohort		PSC: 376 Non-PSC: 162			Cirrhosis: 376 (100%)	
Alkhatib (2011)	Retrospective cohort	PSC: 75	PSC: 185	Mean 50.1 (range 15–85)	58 (77%)	Cirrhosis: 6 (8%)	Autoimmune hepatitis: 2 (3%) IBD: 42 (55%)
Baluyut (2001)	Retrospective cohort	63		Mean 46.7 ± 15.8	38 (60%)	Baseline cholangitis: 31 (49%) Dominant stricture: 63 (100%)	
Bangarulingam (2009)	Retrospective cohort	PSC: 168 Non-PSC: 981	PSC: 308 Non-PSC: 1268	PSC mean: 48 ± 15 Non-PSC mean: 60 ± 19	PSC: 111 (66.1%) Non-PSC: 440 (44.9%)	Portal hypertension: 53 (32%)	
Cazzagon (2019)	Retrospective cohort	PSC: 31	PSC: 63	Median: 36 (27–52)	20 (65%)	Baseline cholangitis: 5 (16%) Cirrhosis: 10 (32%) Dominant stricture: 31 (100%)	
Enns (2003)	Retrospective cohort	PSC: 104	PSC: 204	Mean 45 ± 13.6	66 (63%)	Dominant stricture: 69 (66%)	IBD: 94 (53%)
Etzel (2008)	Retrospective cohort	PSC: 30 Non-PSC: 45	PSC: 85 Non-PSC: 70	PSC mean: 44.5 ± 2.1 (range 24–69) Non-PSC mean: 56.5 ± 2.4 (range 28–85)	PSC: 22 (73.3%) Non-PSC: 28 (62.2%)	Dominant stricture: 63 (76%) Mean duration of PSC: 4.55 years (range 0–15)	
Gluck (2008)	Retrospective cohort	PSC: 106	PSC: 317	Mean: 47 (range 15–86)	65 (61%)	Cirrhosis: 41 (39%)	Cholangiocarcinoma: 13 (12%) IBD: 86 (74%) Liver transplant: 7 (7%)
Gotthardt (2010)	Prospective cohort	PSC: 96	PSC: 500	Mean: 37.4 ± 1.4 (range 17–77)	69 (71.9%)	Dominant stricture: 97 (100%)	Cholangiocarcinoma: 6 (6%) Liver transplant: 22 (23%)
Ismail (2012)	Retrospective cohort	PSC: 441		Median: 38 (range 5–80)	238 (54%)	Prior biliary sphincterotomy: 147 (38%)	
Kaya (2001)	Retrospective cohort	Balloon dilation: 34 Balloon dilation and stenting: 14	Balloon dilation: 73 Balloon dilation and stenting: 80	Balloon dilation median: 50.5 (range 21–72) Balloon dilation and stenting median: 44 (range 18–78)	Balloon dilation: 22 (64.7%) Balloon dilation and stenting: 18 (48.6%)	Dominant stricture: 71 (100%)	IBD: 45 (63%)
Koskensalo (2020)	Retrospective case-control	Diclofenac: 378 No diclofenac: 553	Diclofenac: 1000 No diclofenac: 1000	Diclofenac median: 40 (range 16–73) No diclofenac median: 39 (16–79)	Diclofenac: 238 (63%) No diclofenac: 327 (59.1%)		
Navaneethan (2015)	Retrospective cohort	PSC: 294	PSC: 657	Median: 47 (range 12–85)	203 (69%)	Prior biliary sphincterotomy: 64 (21.8%)	
Navaneethan (2017)	Prospective cohort with the historical control group	Group 1 (control): 156 Group 2 (bile aspiration before contrast injection): 46	Group 1: 156 Group 2: 46	Group 1 mean: 43.77 ± 18.9 Group 2 mean: 54.96 ± 15.8	Group 1: 104 (66.7%) Group 2: 27 (58.7%)	Dominant stricture: 47 (23%) Prior biliary sphincterotomy: 57 (28%) Prior pancreatic sphincterotomy: 1 (0.5%)	
Parlak (2004)	Retrospective cohort	PSC: 16	PSC: 58	Mean: 35 ± 11.2	10 (62.5%)	Dominant stricture: 16 (100%)	IBD: 10 (63%)
Peiseler (2018)	Retrospective cohort	PSC: 208	PSC: 663	Mean: 45.3 (range 25–79)	134 (64.4%)	Cirrhosis: 138 (66%)	IBD: 128 (62%) Immunosuppressed: 94 (45%)
Ponsioen (2018)	Randomized controlled trial		Balloon dilation: 30 Stenting: 33	Mean: 40 ± 14	45 (69.2%)	Dominant stricture: 65 (100%) Median disease duration: 4–7 years Prior sphincterotomy: 25 (39%)	IBD: 50 (78%)
Rupp (2019)	Retrospective cohort	286	>1800	Median: 33.3	209 (73.1%)	Dominant stricture: 179 (63%)	IBD: 209 (73%)
Thiruvengadam (2016)	Retrospective cohort	Rectal indomethacin: 91 No rectal indomethacin: 90					
Von Seth (2015)	Retrospective cohort	PSC: 141 Non-PSC: 8791	PSC: 141 Non-PSC: 8791	PSC mean: 45 ± 16 Non-PSC mean: 69 ± 16	PSC: 87 (62%) Non-PSC: 3868 (44%)	Prior sphincterotomy: 26 (18%)	

*ERCP*, endoscopic retrograde cholangiopancreatography; *IBD*, inflammatory bowel disease; *PSC*, primary sclerosing cholangitis.

**Table 3:** Characteristics of ERCP.

	Precut *n* (%)	Biliary stent insertion *n* (%)	Pancreatic stent insertion *n* (%)	Biliary sphincterotomy *n* (%)	Pancreatic sphincterotomy *n* (%)	Contrast injection into pancreatic duct *n* (%)	Accidental pass into pancreatic duct *n* (%)	Endoscopist experience	Prophylactic antibiotics (%)
Adler (2016)		274 (50.9%)	8 (1.5%)	87 (16.2%)				Experienced interventional endoscopists	Periprocedural (100%)
Alkhatib (2011)		70 (38%)		76 (41%)					Intraprocedural (77%) Postprocedural for 3–7 days (100%)
Baluyut (2001)		33 (52%)		Sphincterotomy NOS: 40 (63%)					Pre- and postprocedural (100%)
Bangarulingam (2009)		PSC: 42 (25%) Non-PSC: 265 (27%)	PSC: 3 (2%) Non-PSC: 129 (13%)	Sphincterotomy NOS PSC: 42 (25%) Non-PSC: 569 (58%)					PSC: Preprocedural (100%) Non-PSC: N/A
Cazzagon (2019)									
Enns (2003)		29 (19.6%)							
Etzel (2008)		PSC: 51 (60.0%) Non-PSC: 60 (85.7%)							PSC: Preprocedural (98.8%) and postprocedural (89.4%) Non-PSC: Preprocedural (95.7%) and postprocedural (58.6%)
Gluck (2008)		43 (51%)		64 (60.4%)					Intraprocedural (100%)
Gotthardt (2010)		5 (5.2%)						Performed by an experienced physician	Periprocedural (100%)
Ismail (2012)	9 (2.0%)			176 (39.9%)	4 (0.91%)	15 (3.4%)	129 (29.3%)	Expert endoscopist	Preprocedural (100%)
Kaya (2001)		Balloon dilation: 0 (0%) Balloon dilation and stenting: 14 (100%)							Balloon dilation alone: Preprocedural (100%) and postprocedural (64.7%) Balloon dilation and stenting: Preprocedural (100%) and postprocedural (51.4%)
Koskensalo (2020)	Diclofenac: 8 (0.8%) No diclofenac: 0 (0%)		Diclofenac: 1 (0.1%) No diclofenac: 0 (0%)	Diclofenac: 215 (21.5%) No diclofenac: 409 (40.9%)			Diclofenac: 90 (9%) No diclofenac: 142 (14.2)		Diclofenac: Preprocedural (100%) No diclofenac: Preprocedural (100%)
Navaneethan (2015)	19 (2.9%)	244 (37.1%)		31 (10.5%)	3 (1.0%)	7 (9.1%)	32 (4.9%)	Experienced interventional endoscopists	Intraprocedural (100%)
Navaneethan (2017)	Group 1 (control): 11 (7.1%) Group 2 (bile aspiration before contrast injection): 2 (4.4%)	Group 1: 83 (53.2%) Group 2: 0 (0%)		Group 1: 4 (2.6%) Group 2: 11 (23.9%)	Group 1: 4 (2.6%) Group 2: 2 (4.4%)	Group 1: 27 (17.3%) Group 2: 5 (10.9%)	Group 1: 15 (9.6%) Group 2: 2 (4.4%)	Experienced interventional endoscopists with a minimum of 1000 ERCPs prior to the study and an annual average of 400 ERCPs	Group 1: intraprocedural (100%) and postprocedural for 5 days (100%) Group 2: N/A
Parlak (2004)		8 (13.8%)		Sphincterotomy NOS: 37 (100%)					
Peiseler (2018)		69 (10.4%)		116 (17%)				Experienced endoscopist	Intraprocedural and postprocedural for 2 days (96%)
Ponsioen (2018)		34 (52.3%)		24 (36.9)					
Rupp (2019)				Sphincterotomy NOS: 286 (100%)				Experienced endoscopists	Prophylactic antibiotics (100%)
Thiruvengadam (2016)								Endoscopists ranged in experience	
Von Seth (2015)	PSC: 15 (11%) Non-PSC: 756 (9%)	PSC: 38 (27%) Non-PSC: 3226 (37%)	PSC: 2 (1.4%) Non-PSC: 63 (0.6%)	Sphincterotomy NOS: PSC: 50 (35%) Non-PSC: 5554 (63%)			PSC: 44 (31%) Non-PSC: 2472 (28%)		PSC: prophylactic (49%) Non-PSC: prophylactic (36%)

*N/A*, not available; *NOS*, not otherwise specified.

**Table 4:** Risk of bias of cohort studies using the Newcastle-Ottawa scale.

	Selection				Comparability	Outcome		
	Representativeness of the exposed cohort	Selection of the nonexposed cohort	Ascertainment of exposure	Demonstration that outcome of interest was not present at start of study	Comparability of cohorts on the basis of the design or analysis	Assessment of outcome	Length of follow-up sufficient for outcomes to occur	Adequacy of follow-up of cohorts
Adler (2016)	—	^*∗*^	^*∗*^	^*∗*^	—	^*∗*^	^*∗*^	—
Bangarulingam (2009)	^*∗*^	^*∗*^	^*∗*^	^*∗*^	—	^*∗*^	^*∗*^	—
Etzel (2008)	^*∗*^	^*∗*^	^*∗*^	^*∗*^	—	^*∗*^	^*∗*^	—
Ismail (2012)	^*∗*^	NR	^*∗*^	^*∗*^	NR	^*∗*^	^*∗*^	—
Navaneethan (2015)	^*∗*^	NR	^*∗*^	^*∗*^	NR	^*∗*^	^*∗*^	^*∗*^
Peiseler (2018)	^*∗*^	^*∗*^	^*∗*^	^*∗*^	^*∗*^	^*∗*^	^*∗*^	^*∗*^
Von Seth (2015)	^*∗*^	^*∗*^	^*∗*^	^*∗*^	–	^*∗*^	^*∗*^	—

*NR*, not relevant. Asterisks indicate the star rating according to the Newcastle-Ottawa scale for cohort studies. A study can be awarded a maximum of 4 stars for selection, 2 stars for comparability, and 3 stars for the outcome.

**Table 5:** Risk of bias of case-control study using the Newcastle-Ottawa scale.

	Selection				Comparability	Exposure		
	Adequacy of case definition	Representativeness of cases	Selection of controls	Definition of controls	Comparability of cohorts on the basis of the design or analysis	Ascertainment of exposure	Same method of ascertainment	Nonresponse rate
Koskensalo (2020)	—	^*∗*^	—	^*∗*^	—	^*∗*^	^*∗*^	NR

*NR*, not relevant. Asterisks indicate the star rating according to the Newcastle-Ottawa scale for cohort studies. A study can be awarded a maximum of 4 stars for selection, 2 stars for comparability, and 3 stars for the outcome.

**Table 6:** Risk of bias of randomized controlled trial using the Cochrane Risk of Bias 2 tool.

	Random sequence generation (selection bias)	Allocation concealment (selection bias)	Blinding of participants and personnel (performance bias)	Blinding of outcome assessment (detection bias)	Incomplete outcome data (attrition bias)	Selective reporting (reporting bias)	Other bias
Ponsioen (2018)	⊕	⊕			⊕	⊕	

The risk of bias assessment for randomized controlled trials is based on the Cochrane Risk of Bias 2 tool. ⊕ indicates that the study has met the domain criterion, while an empty cell indicates that it is unclear whether the domain criterion has been met.

**Table 7:** Summary of results.

30-day ERCP-related adverse events in patients with and without PSC					
Population: adults with PSC Setting: inpatient and outpatient Intervention: ERCP Comparison: adults without PSC					
Outcome	Relative effect (95% CI)	Number of patients (studies)	Sensitivity analysis (95% CI)	Number of patients (studies)	Certainty of the evidence (GRADE)

Cholangitis	OR 3.263 (1.076–9.896), *p*=0.037, *I*^2^ = 73.0%	715 with PSC and 9979 without PSC (4)	OR 5.159 (2.080–12.796), *p* < 0.001, *I*^2^ = 39.9%	339 with PSC and 9817 without PSC (3)	Very low
Pancreatitis	OR 0.888 (CI 0.257–3.069), *p*=0.851, *I*^2^ = 87.9%		OR 1.794 (1.002–3.214), *p*=0.049, *I*^2^ = 29.3%		Very low
Bleeding	OR 0.363 (0.060–2.214), *p*=0.272, *I*^2^ = 50.3%		OR 0.782 (0.207–2.959), *p*=0.718, *I*^2^ = 3.8%		Very low
Perforation	OR 1.191 (0.402–3.515), *p*=0.752, *I*^2^ = 28.5%		OR 1.666 (0.686–4.046), *p*=0.259, *I*^2^ = 0.0%		Very low

Risk factors for 30-day post-ERCP pancreatitis					
Population: adults with PSC Setting: inpatient and outpatient Intervention: ERCP Comparison: N/A					

Risk factor	Relative effect (95% CI)	Number of procedures (studies)	Certainty of the evidence (GRADE)		

Female sex	OR 1.546 (0.882–2.709), *p*=0.128, *I*^2^ = 47.6%	3824 (5)	Very low		
Accidental passage of wire into the pancreatic duct	OR 7.444 (3.328–16.651), *p* < 0.001, *I*^2^ = 65.0%	3098 (3)	Very low		
Biliary sphincterotomy	OR 4.802 (1.916–12.033), *p*=0.001, *I*^2^ = 73.1%	3824 (5)	Very low		

*CI*, confidence interval; *ERCP*, endoscopic retrograde cholangiopancreatography; *GRADE*, Grading of Recommendations, Assessment, Development, and Evaluations; *N/A*, not applicable; *OR*, odds ratio; *PSC*, primary sclerosing cholangitis.

## Data Availability

Additional data are available in supplementary appendices. Further data can be obtained from the corresponding author upon request.

## Supplementary Materials

Supplementary Materials Supplemental Figure S1. PRISMA guideline checklist. Supplemental Figure S2. Embase search strategy. Supplemental Figure S3. Sensitivity analysis of post-ERCP adverse events.
